# Trends and outcomes of neoadjuvant radiotherapy compared with postoperative radiotherapy for malignant breast cancer

**DOI:** 10.18632/oncotarget.24313

**Published:** 2018-05-11

**Authors:** Wenyan Fu, Hefen Sun, Yang Zhao, Mengting Chen, Lipeng Yang, Shuiping Gao, Liangdong Li, Wei Jin

**Affiliations:** ^1^ Department of Breast Surgery, Key Laboratory of Breast Cancer in Shanghai, Collaborative Innovation Center of Cancer Medicine, Fudan University Shanghai Cancer Center, Shanghai 200030, China; ^2^ Department of Oncology, Shanghai Medical College, Fudan University, Shanghai 200030, China; ^3^ Department of Pathology, School of Basic Medical Sciences, Fudan University, Shanghai 200030, China

**Keywords:** breast cancer, radiotherapy, radiation sequence with surgery, survival rate, SEER

## Abstract

**Background:**

Although neoadjuvant treatment has become the established approach for women with large primary tumors or locally advanced breast cancer for which immediate surgery is not the best approach, it may also stimulate cancer stem cell self-renewal and facilitate recurrence. We sought to determine the survival outcomes of preoperative radiotherapy (PRRT) compared with postoperative radiotherapy (PORT).

**Materials and Methods:**

The Surveillance, Epidemiology, and End Results (SEER) registry was queried for patients who were diagnosed with breast cancer and underwent cancer-directed surgery. Survival analyses were performed with Cox proportional hazard regression for both overall survival (OS) and disease-specific survival (DSS), and 1:1 propensity score (PS) matching-adjusted competing risk analyses were conducted for DSS.

**Results:**

We first identified 1,111,218 eligible patients in 18 registries from 1973 to 2013 and found that, outside of the Utah registry, sequence patterns other than PORT were rarely used. Thus, we next identified eligible patients registered in Utah (*n* = 7,042) from 1988 to 2007. The treatment trends shifted abruptly in 1988. Compared with the PORT group, the PRRT group showed significantly higher risks of overall mortality (absolute difference, 22.4%; *P* < 0.001), breast cancer-specific mortality (absolute difference, 8.6%; *P* < 0.001), and cardiovascular disease-specific mortality (absolute difference, 11.5%; *P* = 0.021). Survival differences in treatment sequences were correlated with stage.

**Conclusions:**

Substantial shifts in treatment patterns for malignant breast cancer were identified in Utah. Compared with PORT, PRRT showed significantly worse outcomes. These results could inform future standardized options for radiation sequence with surgery and further clinical trials.

## INTRODUCTION

Breast cancer is the most prevalent cancer diagnosis and the leading cause of cancer-related death among females worldwide, with an estimated 1.7 million cases and 521,900 deaths reported in 2012 [1]. With the increased understanding and molecular characterization of breast cancer, surgery, radiotherapy and systemic treatments, particularly cytotoxic chemotherapy and, more recently, molecular-targeted agents, are the current mainstay of clinical cancer care. These treatments not only improve local tumor control but also sometimes eradicate micro-metastasis, thereby improving survival.

Despite continuous improvements in cancer management, local-regional recurrence or metastatic spread still occurs in a high proportion of patients [2, 3]. At least for radiotherapy and systemic treatments, one underlying reason for these occurrences might be the low efficacy of current treatments for eradicating cancer stem cells (CSCs). One of the first observations of CSCs was published by Dr. Richards in 1955. He demonstrated the existence of a stem cell pool using deoxyribose nucleic acid measurements in Ehrlich and Krebs ascites tumors. He observed that only a small fraction of cells was capable of normal and regular mitosis and that this finding could be due to stem cell division [4]. The cancer stem cell model argues that stem cells are the major contributing factor to disease progression and therapy response, as they have the capability to self-renew and generate heterogeneous lineages of cancer cells [5]. The CSC hypothesis has important implications for therapeutic development. CSCs are relatively resistant to cytotoxic chemotherapy, both in preclinical models and in neoadjuvant clinical trials [6].

Currently, sequence patterns of radiotherapy (RT) with surgery mainly include radiation prior to surgery, intraoperative radiation, and radiation after surgery. Postoperative RT (PORT) has long been recognized as a key component of breast-conserving surgery (BCS) and has been recommended in consensus guidelines as a standard surgical option for over 2 decades [7, 8]. Additionally, there are consensus guidelines for selecting post-mastectomy RT among patients with operable invasive breast cancer [8]. Large randomized trials and statistical studies have established the benefit of PORT in breast cancer, with improved survival and reduced rate of local recurrence in appropriately selected patients [9–11]. Preoperative RT (PRRT), similar to neoadjuvant chemotherapy, may be applicable not only for patients with locally advanced breast cancer and inoperable advanced-stage tumors for BCS but also for those with operable tumors, aiming at breast conservation and higher complete remission rates [12, 13]. However, neoadjuvant treatment may also stimulate CSC self-renewal through cytokine production and DNA repair mechanisms. Therefore, the question concerning the impact of PRRT on long-term survival is important and remains to be answered.

In this study, we utilized a population-based study to examine the overall outcomes of radiotherapy with surgery in different sequence patterns. We investigated the association of different radiation sequences with surgery and survival outcomes of malignant breast cancer; furthermore, we assessed the results based on data stratified by stage, a critical clinical index of surgery, and clinicopathologic factors associated with local recurrence risk and long-time survival.

## MATERIALS AND METHODS

### Ethics statement

This study was approved by the Fudan University Shanghai Cancer Center Ethics Committee after undergoing an independent ethics committee/institutional review. Data from the National Cancer Institute's Surveillance, Epidemiology, and End Results (SEER) database were obtained online. Written informed consent was obtained from the patient for publication of this report and any accompanying images.

### Study design and data source

The SEER database [14], which represents almost 30% of all new cancer diagnoses per year, is one of the largest validated cancer registries in the United States. In the present study, a retrospective cohort study was conducted with data obtained from SEER. We identified 1,111,218 females greater than or equal to age of 20 who underwent cancer-directed surgery after being diagnosed with a first case of breast cancer using the SEER program, which included 18 population-based cancer registries dating from 1973 to 2013 (from the first registry to the last update in SEER database). Cases diagnosed by autopsy or death certificate only or with site-specific surgery codes of 0 or 9 or primary site-surgery codes of 0 or 99 were excluded from the analysis. Among the remaining patients, 636,181 from 18 registries and 7,042 from the Utah registry in 1988–2007 were selected to investigate the trends and outcomes of cancer-directed surgery with and without radiation and with different surgery-radiation sequences. To acquire more comprehensive information on stage and regional node status, patients with breast cancer diagnosed before 1988 were excluded. Moreover, those diagnosed after 2007 were excluded due to inadequate follow-up time. Additionally, all of the 1,111,218 cases were included in the analysis of overall rates of radiation sequence with surgery.

### SEER treatment variables and covariates

In our study, Hispanic origin, race (white, black, others/unknown), age (20–39 years, 40–49 years, 50–69 years, ≥70 years), place of residence (large urban, small urban, rural), and marital status (married, not married, unknown) were included as patient-specific variables. Based on previous reports [10, 15], we divided age of patients by less than 50, 50–69 and greater than 70. Since breast cancer in young adult less than age of 40 is one special type, which has higher proportions of high-grade and later-stage tumors; a more-aggressive phenotype and worse prognosis [16], we further divided the group of less than 50 into two groups of 20–39 and 40–49 for better understanding of outcomes stratified by the radiation sequence with surgery. Grade (high, intermediate, low, unknown), laterality (unilateral, bilateral), American Joint Committee on Cancer (AJCC) 6th stage (I, II, III, IV, others/unknown), lymph node (LN) status (negative, positive, unknown), estrogen receptor (ER) status (positive, negative, borderline, unknown), progesterone receptor (PR) status (positive, negative, borderline, unknown), tumor size (≤20 mm, 21–50 mm, >50 mm, unknown), and surgery mode (breast-conserving surgery [BCS], mastectomy, others/unknown) were included as tumor-specific variables. All variables were categorized as outlined in Supplementary Table 1. The procedure was repeated for 5 cycles to produce a single imputed dataset to stabilize the results. For analysis, the classification of all variables remained consistent in this study to allow for a nonlinear effect in regression models. We applied surgery-radiation sequence codes to classify patients with the “No radiation and/or cancer-directed surgery” code into the surgery-alone group (non-RT group) and those with other codes into the surgery with radiation group (RT group).

### Outcome measurement

Cause of death was categorized as breast cancer, other malignant disease, cardiovascular disease, infectious disease, pulmonary disease, diabetes mellitus, or other/unknown according to the primary cause of death listed in SEER and on the basis of the International Classification of Diseases Revisions 8 to 10. Time to overall death and breast cancer-specific death (overall mortality [OM] and breast cancer-specific mortality [BCM]) was calculated based on the time period from the date of diagnosis until the last date for which complete vital status data were available (last follow-up date: December 31, 2013). Data regarding deaths were ascertained from death certificates, which are coded by state health departments and/or state vital records for each SEER region [17].

### Statistical analysis

For categorical (counts, percentage) variables, demographic and tumor-specific characteristics were compared between the different treatment groups using Pearson's Chi-square test. The Cochrane Armitage trend test was used to assess treatment shifts over time, and Cox proportional hazard regression analysis was used to evaluate overall survival (OS) and disease-specific survival (DSS). In the survival analysis, all of the patients lost to follow-up were censored. In our study, multivariable analysis as well as 1:1 propensity-score (PS) matching, which aims to reduce the effect of treatment selection by creating a pseudo-randomized study design based on all measured potential confounders in the dataset, were applied to adjust for potentially indicated confounders (covariates: age, marital status, race, place of residence, Hispanic origin, laterality, tumor grade, stage, LN status, surgery mode, ER status, and PR status), as the treatment assignment of patients was not random (Table 2) [18]. Psmatching3 in SPSS was used for PS matching and to test matching quality to determine the balance after the match [19]. The effects of individual variables on DSS and OS with and without PS matching were further calculated. We used the Kaplan-Meier method to generate survival curves and log-rank tests to analyze the differences between curves. Additional stratified analyses were conducted using four stage groups (stage I, stage II, stage III, and stage IV). In addition, we performed a secondary analysis using 1:1 PS matching-adjusted competing risk analyses to confirm the HRs for disease-specific mortality, which adjusted for competing events such as death from other non-cancer causes or death from unknown reasons [20].

All statistical analyses were performed using SPSS statistical software, version 22.0 (IBM Corp, Armonk, NY) or R version 3.2.3 [21]. All statistical testes were two-sided, with statistical significance determined by *P* values below 0.05.

## RESULTS

### Survival benefit of adjuvant radiation with cancer-directed surgery

We identified 636,181 patients with malignant breast cancer from 18 registries dating from 1988 to 2007 (Supplementary Table 1). Of this cohort, 351,768 patients were stratified into the non-RT group and 284,413 patients were stratified into the RT group. The largest proportion of patients in the non-RT group were those who underwent mastectomy (72.3%), whereas most of the patients in the RT group were treated with BCS (80.2%) (Supplementary Table 1). However, an increase in the use of radiation with surgery was observed over time among all patient groups, including both the BCS group (from 68.3% in 1988–1992 to 70.6% in 2003–2007, *P*_trend_ < 0.001) and the mastectomy group (from 9.8% in 1988–1992 to 22.3% in 2003–2007, *P*_trend_ < 0.001). Overall, the rate increased from 29.0% in 1988–1992 to 49.3% in 2003–2007 (*P*_trend_ < 0.001) (Table 1).

**Table 1:** Trends of patient characteristics undergoing surgery with RT compared with surgery alone in 18 registries from 1988–2007^*^

	No. of Patients (%)				
Characteristic	Number of patients who underwent surgery with RT (% of all surgery patients)				*P*_trend_
Year of diagnosis	1988–1992	1993–1997	1998–2002	2003–2007	
Number of patients	22697 (29.0)	45164 (41.2)	94504 (47.1)	122048 (49.3)	<.001
Race					
White	19824 (29.1)	38432 (41.6)	80361 (47.6)	101556 (49.6)	<.001
Black	1638 (28.1)	3376 (37.5)	7549 (42.8)	11313 (47.3)	<.001
Other/unknown	1235 (28.8)	3356 (40.6)	6594 (46.7)	9179 (48.3)	<.001
Hispanic					
Yes	1078 (30.7)	3035 (40.6)	6927 (45.7)	10557 (47.8)	<.001
No	21619 (28.9)	42129 (41.2)	87577 (47.2)	111491 (49.5)	<.001
Age group, y					
20–39	1924 (36.2)	3044 (44.5)	5609 (50.1)	6762 (50.9)	<.001
40–49	4647 (35.3)	9143 (46.5)	18174 (51.4)	24177 (52.5)	<.001
50–69	10755 (32.1)	21444 (45.8)	46912 (51.7)	62530 (53.8)	<.001
≥70	5371 (20.4)	11533 (31.7)	23809 (37.5)	28579 (39.8)	<.001
Married					
Yes	13746 (31.7)	26946 (44.6)	56753 (50.9)	73022 (52.8)	<.001
No	8495 (25.8)	17369 (37.3)	35110 (43.0)	45833 (45.5)	<.001
Unknown	456 (22.7)	849 (30.8)	2641 (35.5)	3193 (38.1)	<.001
Laterality					
Unilateral	22672 (29.0)	45138 (41.2)	94453 (47.1)	121998 (49.3)	<.001
Bilateral	25 (19.1)	26 (22.8)	51 (25.8)	50 (24.6)	0.541
Grade					
High	1873 (36.3)	7170 (47.4)	19481 (51.9)	26266 (52.9)	<.001
Intermediate	5595 (31.8)	16220 (43.4)	36206 (47.7)	48098 (50.0)	<.001
Low	6127 (30.5)	14654 (40.2)	31187 (46.4)	40824 (48.4)	<.001
Unknown	9102 (25.6)	7120 (34.3)	7630 (38.4)	6860 (39.5)	<.001
Stage					
I	10672 (33.0)	23234 (47.8)	48334 (51.2)	62448 (52.8)	<.001
II	5336 (23.6)	10946 (35.0)	26934 (41.9)	36562 (43.9)	<.001
III	3579 (33.3)	5907 (42.0)	13876 (54.9)	17996 (59.0)	<.001
IV	777 (35.5)	883 (38.9)	1453 (38.6)	1844 (38.0)	0.078
Unknown/other	2333 (22.3)	4194 (31.2)	3907 (30.1)	3198 (29.8)	<.001
LN status					
Negative	12650 (28.9)	25782 (43.8)	55628 (49.3)	76639 (51.9)	<.001
Positive	6571 (28.7)	11624 (38.7)	30692 (49.1)	39499 (51.1)	<.001
Unknown	3476 (29.8)	7758 (37.3)	8184 (32.4)	5910 (26.2)	<.001
ER status					
Positive	9676 (33.3)	29385 (44.3)	65994 (50.7)	91471 (51.8)	<.001
Negative	3001 (33.2)	8758 (41.6)	16912 (47.6)	24018 (48.6)	<.001
borderline	265 (31.9)	241 (38.1)	148 (42.4)	232 (40.6)	<.001
Unknown/other	9755 (24.7)	6780 (31.3)	11450 (33.2)	6327 (30.1)	<.001
PR status					
Positive	8345 (33.9)	25085 (44.5)	55439 (51.4)	77051 (52.4)	<.001
Negative	4045 (32.3)	11739 (42.0)	25488 (47.8)	36697 (49.0)	<.001
borderline	182 (26.4)	344 (36.8)	449 (45.2)	876 (45.7)	<.001
Unknown/other	10125 (25.0)	7996 (32.8)	13128 (34.2)	7424 (31.2)	<.001
Surgery, primary site					
BCS	17489 (68.3)	37265 (71.3)	75595 (70.0)	97670 (70.6)	<.001
Mastectomy	5135 (9.8)	7740 (13.6)	18770 (20.4)	24254 (22.3)	<.001
Unknown/other	73 (21.0)	159 (30.1)	139 (33.3)	124 (27.9)	0.002
Living					
Urban large	18990 (30.3)	38310 (41.9)	77470 (47.9)	99325 (50.3)	<.001
Urban small	2742 (23.5)	4765 (36.0)	14569 (43.9)	20247 (45.6)	<.001
Rural	183 (17.0)	393 (29.1)	1434 (36.9)	2345 (42.3)	<.001
Unknown	782 (27.7)	1696 (46.5)	1031 (52.9)	131 (39.6)	<.001

^*^RT = radiotherapy; LN = Lymph node; ER = estrogen receptor, PR = progesterone receptor; BCS = Breast-conserving surgery.

Survival analyses were performed between the non-RT and RT groups, with a median follow-up time of 109 months for patients with malignant breast cancer (interquartile range [IQR], 74–154 months). As a result, patients who received radiation with surgery had better OS (hazard ratio [HR] = 0.79, 95% confidence interval [CI] = 0.79–0.80, *P* < 0.001) and breast cancer-specific survival (BCSS) (HR = 0.90, 95% CI = 0.88 to 0.91, *P* < 0.001) than their counterparts (Supplementary Figure 1). Given that surgery with radiation had greater survival benefit than surgery alone for patients with breast cancer, we further investigated whether radiation sequence with surgery was associated with survival outcomes.

### Trends in radiation sequence with surgery

Next, we identified all of the eligible patients (*n* = 1,111,218) in the 18 registries from 1973 to 2013, stratified them into eight groups according to the sequences recorded, and analyzed the rate of sequences in each registry. The data showed that about half of the patients underwent radiation with surgery, and almost all of the patients received PORT; with the exception of Utah, less than one percent of patients in the registries received other sequences. In Utah, 12.9% of cases were treated with PPRT and 27.4% with PORT (Supplementary Table 2).

Hence, we selected out the patients (*n* = 7,042) registered in Utah from 1988 to 2007 to further analyze the differences in trends and outcomes between PRRT (*n* = 2,371) and PORT (*n* = 4,671). Patient clinicopathologic factors and the balance in patient characteristics achieved after multiple imputations and 1:1 (PRRT: PORT) PS matching adjustments to estimate the average treatment effects are listed in Table 2. Most clinicopathologic factors were related to the sequence of radiation and surgery. Patients diagnosed during later years, younger patients, married patients, patients with low-stage cancer, patients with positive LN status, patients with positive ER/PR status, and patients living in smaller urban regions were less likely to receive PRRT (*P* < 0.05). Between 1988 and 2007, a significant change in treatment sequences was observed. Nearly all of the patients diagnosed between 1988 and 1997 (median percent = 98.8%, IQR = 98.3%-99.1%) underwent PRRT, whereas most of those diagnosed between 1998 and 2007 (median percent = 99.0%, IQR = 97.0%–99.5%) received PORT (Supplementary Figure 2). Moreover, the treatment choice shifted from 1997 (98.0% PRRT) to 1998 (99.4% PORT).

**Table 2:** Characteristics of patients in Utah undergoing PRRT or PORT before and after PS matching adjustment from 1988–2007^*^

	Before PS matching adjustment			After PS matching adjustment		
Characteristic	PRRT No. (%)	PORT No. (%)	*P*-value^†^	PRRT No. (%)	PORT No. (%)	*P*-value^†^
Number of patients	2371	4671		1854	1854	
Race						
White	2331 (98.3)	4559 (97.6)	0.151	1818 (98.1)	1821 (98.2)	0.525
Black	5 (0.2)	13 (0.3)		5 (0.3)	2 (0.1)	
Other/unknown	35 (1.5)	99 (2.1)		31 (1.7)	31 (1.7)	
Hispanic						
Yes	110 (4.6)	204 (4.4)	0.601	96 (5.2)	90 (4.9)	0.652
No	2261 (95.4)	4467 (95.6)		1758 (94.8)	1764 (95.1)	
Age group, y						
20–39	158 (6.7)	285 (6.1)	<.001	132 (7.1)	113 (6.1)	0.589
40–49	379 (16.0)	898 (19.2)		313 (16.9)	311 (16.8)	
50–69	1171 (49.4)	2347 (50.2)		882 (47.6)	909 (49.0)	
≥70	663 (28.0)	1141 (24.4)		527 (28.4)	521 (28.1)	
Married						
Yes	1564 (66.0)	3227 (69.1)	0.003	1186 (64.0)	1222 (65.9)	0.334
No	780 (32.9)	1367 (29.3)		649 (35.0)	609 (32.8)	
Unknown	27 (1.1)	77 (1.6)		19 (1.0)	23 (1.2)	
Laterality						
Unilateral	2369 (99.9)	4670 (100.0)	0.226	1853 (99.9)	1853 (99.9)	1.000
Bilateral	2 (0.1)	1 (0.0)		1 (0.1)	1 (0.1)	
Grade						
High	399 (16.8)	940 (20.1)	<.001	327 (17.6)	346 (18.7)	0.451
Intermediate	956 (40.3)	2018 (43.2)		826 (44.6)	786 (42.4)	
Low	560 (23.6	1486 (31.8)		467 (25.2)	497 (26.8)	
Unknown	456 (19.2)	227 (4.9)		234 (12.6)	225 (12.1)	
Stage						
I	1201 (50.7)	2283 (48.9)	<.001	989 (53.3)	931 (50.2)	0.368
II	513 (21.6)	1381 (29.6)		433 (23.4)	447 (24.1)	
III	329 (13.9)	774 (16.6)		248 (13.4)	280 (15.1)	
IV	59 (2.5)	70 (1.5)		42 (2.3)	45 (2.4)	
Unknown/other	269 (11.3)	163 (3.5)		142 (7.7)	151 (8.1)	
LN status						
Negative	1453 (61.3)	2788 (59.7)	<.001	1189 (64.1)	1139 (61.4)	0.235
Positive	645 (27.2)	1676 (35.9)		507 (27.3)	547 (29.5)	
Unknown	273 (11.5)	207 (4.4)		158 (8.5)	168 (9.1)	
ER status						
Positive	1396 (58.9)	3548 (76.0)	<.001	1323 (71.4)	1356 (73.1)	0.460
Negative	347 (14.6)	953 (20.4)		339 (18.3)	329 (17.7)	
borderline	20 (0.8)	18 (0.4)		16 (0.9)	18 (1.0)	
Unknown/other	608 (25.6)	152 (3.3)		176 (9.5)	151 (8.1)	
PR status						
Positive	1227 (51.8)	3159 (67.6)	<.001	1155 (62.3)	1200 (64.7)	0.327
Negative	491 (20.7)	1284 (27.5)		484 (26.1)	468 (25.2)	
borderline	31 (1.3)	72 (1.5)		31 (1.7)	30 (1.6)	
Unknown/other	622 (26.2)	156 (3.3)		184 (9.9)	156 (8.4)	
Surgery, primary site						
BCS	1837 (77.5)	3478 (74.5)	0.013	1457 (78.6)	1408 (75.9)	0.158
Mastectomy	531 (22.4)	1190 (25.5)		396 (21.4)	445 (24.0)	
Unknown/other	3 (0.1)	3 (0.1)		1 (0.1)	1 (0.1)	
Living						
Urban large	1920 (81.0)	3621 (77.5)	0.002	1499 (80.9)	1480 (79.8)	0.555
Urban small	344 (14.5)	824 (17.6)		262 (14.1)	285 (15.4)	
Rural	107 (4.5)	226 (4.8)		93 (5.0)	89 (4.8)	
Year^‡^						
1988–1992	854 (36.0)	14 (0.3)	<.001	528 (28.5)	10 (0.5)	<.001^§^
1993–1997	1368 (57.7)	15 (0.3)		1185 (63.9)	11 (0.6)	
1998–2002	12 (0.5)	2345 (50.2)		11 (0.6)	973 (52.5)	
2003–2007	137 (5.8)	2297 (49.2)		130 (7.0)	860 (46.4)	
Status						
Dead breast	481 (20.3)	632 (13.5)	<.001	374 (20.2)	250 (13.5)	<.001^§^
Dead other	833 (35.1)	680 (14.6)		629 (33.9)	311 (16.8)	
Alive	1057 (44.6)	3359 (71.9)		851 (45.9)	1293 (69.7)	

^*^Numbers before (*n =* 7042 patients) and after (*n =* 3720 patients) adjustment differ with 1:1 propensity score (PS) matching. Covariates used for PS matching-adjusted analysis: age, race, Hispanic origin, marital status, place of residence, laterality, tumor grade, stage, LN status, surgery mode, ER status, and PR status. LN = Lymph node; ER = estrogen receptor; PR = progesterone receptor; PRRT = preoperative radiotherapy; PORT = postoperative radiotherapy.

^§^Not applicable (patients excluded from Multivariable-adjusted analysis).

^†^*P* values based on Pearson's Chi-square test for categorical (counts, percentage) variables.

^‡^Numbers and row percentages.

### Survival benefit of radiation after surgery over radiation prior to surgery

Outcomes of PRRT and PORT for patients with malignant breast cancer were evaluated using survival analyses based on patients in Utah from 1988 to 2007. The median follow-up time of patients from diagnosis was 120 months (IQR = 80–168). In the overall cohort, breast cancer was identified as the predominant cause of death in 15.8% of women, followed by cardiovascular disease (7.3% of all deaths) and unknown causes (7.4% of all deaths). After that, mortality caused by other malignant disease and pulmonary disease accounted for 4.1% and 1.5% of all deaths, respectively (Table 3 shows data for the original patient cohort and the cohort adjusted by PS matching). OS adjusted by PS matching was higher for patients who underwent PORT (5-year OS = 83.8%, 10-year OS = 56.1%) than those who received PRRT (5-year OS = 79.7%, 10-year OS = 33.7%); this finding was confirmed by both multivariable-adjusted (HR = 0.82, 95%CI = 0.75–0.90, *P* < 0.001) and PS matching-adjusted (HR = 0.79, 95%CI = 0.71–0.88, *P* < 0.001) analyses. After PS-matching adjustment, 10-year BCSS was 81.4% in the PORT group and 72.8% in the PRRT group (absolute difference, 8.6%). This result significantly differed from the proportional-hazard assumption in the Cox regression hazard model not only after multivariable adjustment (HR = 0.71, 95%CI = 0.62–0.81, *P* < 0.001) but also after PS-matching adjustment (HR = 0.69, 95%CI = 0.59–0.82, *P* < 0.001) (Table 4; data for other clinical factors showing an effect on OM or BCM are listed in Supplementary Tables 3 and 4). Additionally, a higher risk of cardiovascular disease-specific mortality was detected in patients who underwent PRRT compared to those who underwent PORT (PS matching-adjustment: HR = 0.73, 95%CI = 0.57–0.95, *P* = 0.021). No significant difference in death caused by other malignant disease was observed in the unadjusted groups (*P* = 0.603) or adjusted groups (PS matching-adjustment, *P* = 0.828) (Table 4).

**Table 3:** Causes of mortality stratified by treatment groups in Utah form 1988–2007^*^

	Unadjusted			PS matching-adjusted		
Causes of Death	PRRT No. (%)	PORT No. (%)	Total No. (%)	PRRT No. (%)	PORT No. (%)	Total No. (%)
Number of patients	2371	4671	7042	1854	1854	3708
Alive at last follow-up	1057 (44.6)	3359 (71.9)	4416 (62.7)	851 (45.9)	1293 (69.7)	2144 (57.8)
Cause of death, %						
Breast cancer	481 (20.3)	632 (13.5)	1113 (15.8)	374 (20.2)	250 (13.5)	624 (16.8)
Other malignant disease	143 (6.0)	148 (3.2)	291 (4.1)	107 (5.8)	64 (3.5)	171 (4.6)
Cardiovascular disease	308 (13.0)	208 (4.5)	516 (7.3)	228 (12.3)	98 (5.3)	326 (8.8)
Pulmonary disease	62 (2.6)	45 (1.0)	107 (1.5)	50 (2.7)	19 (1.0)	69 (1.9)
Infectious disease	17 (0.7)	11 (0.2)	28 (0.4)	14 (0.8)	5 (0.3)	19 (0.5)
Diabetes mellitus	24 (1.0)	24 (0.5)	48 (0.7)	18 (1.0)	11 (0.6)	29 (0.8)
Other/unknown	279 (11.8)	244 (5.2)	523 (7.4)	212 (11.4)	114 (6.1)	326 (8.8)

^*^PS: propensity score; PRRT = preoperative radiotherapy; PORT = postoperative radiotherapy.

**Table 4:** Analysis of OS and DSS between groups (unadjusted, multivariable-adjusted, and PS matching-adjusted Cox proportional hazards regression models) in Utah form 1988–2007^*^

Treatment	Unadjusted		Multivariable-adjusted		PS matching-adjusted^†^		PS matching-adjusted survival rates	
	HR (95% CI)	*P*	HR (95% CI)	*P*	HR (95% CI)	*P*	5-year (%)	10-year (%)
Overall survival								
PRRT	Ref.		Ref.		Ref.		79.7	33.7
PORT	0.79 (0.73–0.86)	<.001	0.82 (0.75–0.90)	<.001	0.79 (0.71–0.88)	<.001	83.8	56.1
Breast cancer–specific survival								
PRRT	Ref.		Ref.		Ref.		87.9	72.8
PORT	0.80 (0.71–0.91)	<.001	0.71 (0.62–0.81)	<.001	0.69 (0.59–0. 82)	<.001	90.5	81.4
Other malignant disease–specific survival								
PRRT	Ref.		Ref.		Ref.		98.2	88.4
PORT	0.94 (0.73–1.20)	0.603	0.99 (0.77–1.28)	0.936	0.96 (0.69–1.34)	0.828	97.9	93.2
Cardiovascular disease–specific survival								
PRRT	Ref.		Ref.		Ref.		95.8	77.4
PORT	0.61 (0.51–0.74)	<.001	0.93 (0.43–1.97)	0.839	0.73 (0.57–0.95)	0.021	97.1	88.9

^*^Cox proportional hazard regression analysis for overall survival and competing risk survival models for breast cancer-specific survival were used (two-sided). Covariates for multivariable/PS matching-adjustment: age, race, Hispanic origin, marital status, place of residence, laterality, tumor grade, stage, LN status, surgery mode, ER status, and PR status. CI = confidence interval; HR = hazard ratio; PS = propensity score; PRRT = preoperative radiotherapy; PORT = postoperative radiotherapy; Ref. = referent; OS = overall survival; DSS = disease-specific survival.

^†^Multivariate analysis of PS matching-adjusted groups.

**Figure 1:**
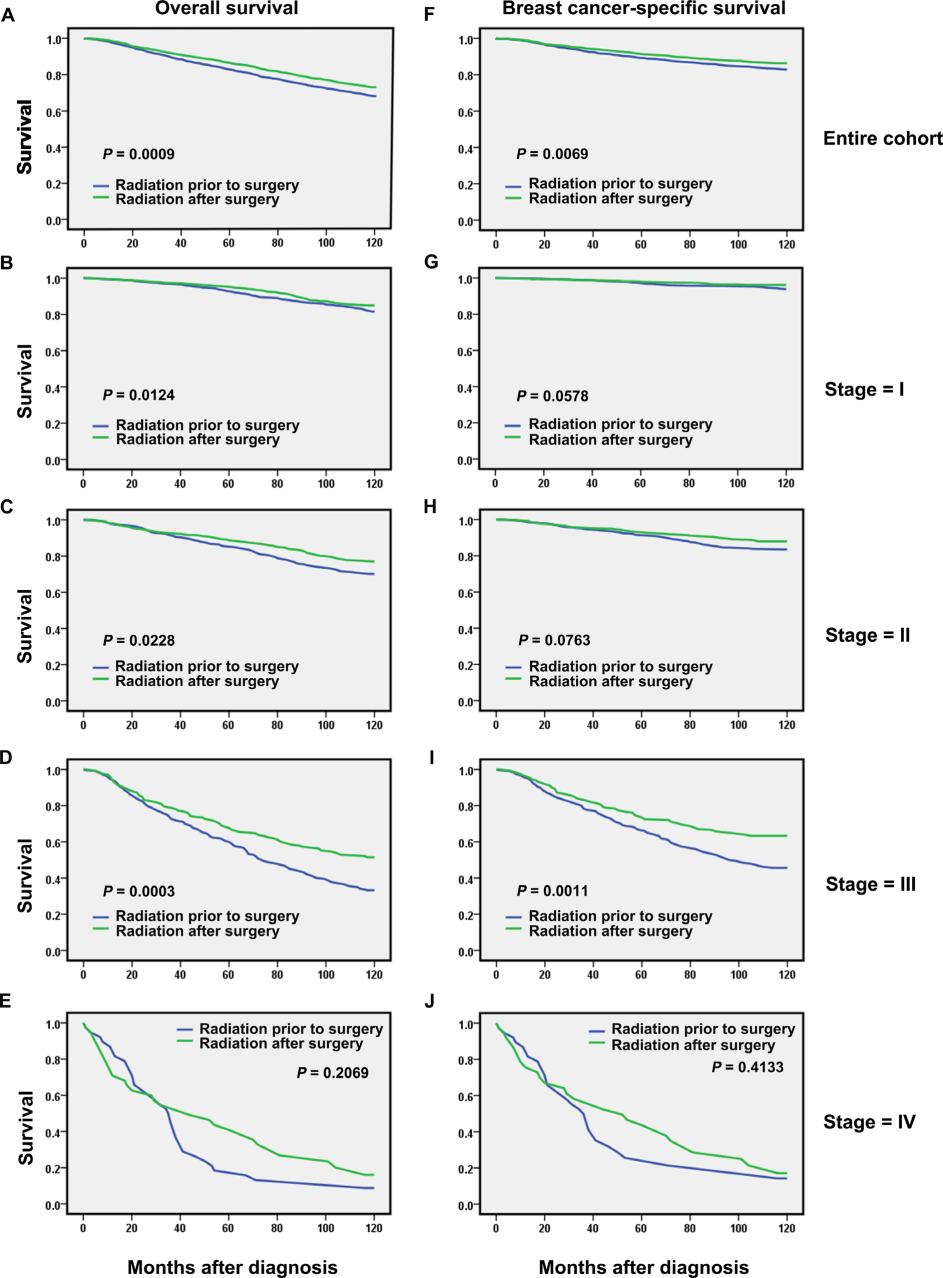
Overall and breast cancer–specific survival by radiation sequence with surgery in the overall group and stratified by stage groups based on propensity score (PS) matching-adjusted survival data in Utah from 1988–2007 All *P* values based on PS matching-adjusted, two-sided log-rank test. Left column, overall survival: (**A**) entire cohort, (**B**) stage I, (**C**) stage II, (**D**) stage III, and (**E**) stage IV. Right column, breast cancer–specific mortality: (**F**) entire cohort, (**G**) stage I, (**H**) stage II, (**I**) stage III, and (**J**) stage IV.

Because the largest proportion of overall mortality was due to breast cancer mortality, we further stratified by AJCC stage, one of the major indices for breast cancer-directed surgery in the NCCN treatment guidelines, and analyzed OS and BCSS in patients from the two groups. Significant correlations of the PS matching-adjusted OS and BCSS with AJCC stages were observed; specifically, patients diagnosed at stage IV demonstrated no significant difference in either 10-year OS (absolute difference, 0.5%; log-rank test, *P* = 0.2069) or 10-year BCSS (absolute difference, 8.5%; log-rank test, *P* = 0.4133) compared with patients diagnosed at stage III, who exhibited marked increases in OS (absolute difference, 25.4%; log-rank test, *P* = 0.0003,) and BCSS (absolute difference, 27.4%; log-rank test, *P* = 0.0011; Figure 1, Table 5). Moreover, patients of stage I and II had a significant increase in 10-year OS (stage I: absolute difference, 27.7%; log-rank test, *P* = 0.0124; stage II: absolute difference, 20.6%; log-rank test, *P* = 0.0228, respectively) but not BCS (stage I: absolute difference, 6.1%; log-rank test, *P* = 0.0578; stage II: absolute difference, 8.4%; log-rank test, *P* = 0.0763, respectively). These results were confirmed by Cox proportional-hazard regression analyses (Table 5).

**Table 5:** Subgroup analysis of overall and breast cancer-specific survival, stratified by 4 stage-groups in Utah form 1988–2007^*^

Treatment	Unadjusted		Multivariable-adjusted		PS matching-adjusted^†^		PS matching-adjusted survival rates	
	HR (95% CI)	*P*	HR (95% CI)	*P*	HR (95% CI)	*P*	5-year (%)	10-year (%)
Overall survival								
Stage I								
PRRT	Ref.		Ref.		Ref.		90.2	42.1
PORT	0.89 (0.77–1.02)	0.097	0.85 (0.74–0.98)	0.028	0.78 (0.65–0.95)	0.011	93.5	69.8
Stage II								
PRRT	Ref.		Ref.		Ref.		81.9	39.7
PORT	0.79 (0.67–0.94)	0.007	0.82 (0.68–0.98)	0.029	0.76 (0.60–1.66)	0.030	87.0	60.3
Stage III								
PRRT	Ref.		Ref.		Ref.		52.1	12.1
PORT	0.60 (0.51–0.71)	<.001	0.64 (0.53–0.77)	<.001	0.62 (0.49–0.79)	<.001	64.7	37.5
Stage IV								
PRRT	Ref.		Ref.		Ref.		13.2	2.9
PORT	1.02 (0.71–1.47)	0.920	0.94 (0.61–1.44)	0.771	0.83 (0.50–1.37)	0.469	35.3	2.4
Breast cancer–specific survival								
Stage I								
PRRT	Ref.		Ref.		Ref.		96.1	85.6
PORT	0.74 (0.56–0.99)	0.040	0.76 (0.56–1.02)	0.067	0.75 (0.52–1.09)	0.137	97.6	91.7
Stage II								
PRRT	Ref.		Ref.		Ref.		89.7	72.1
PORT	0.78 (0.61–1.00)	0.048	0.79 (0.61–1.03)	0.083	0.79 (0.56–1.12)	0.190	91.9	80.5
Stage III								
PRRT	Ref.		Ref.		Ref.		60.9	29.1
PORT	0.65 (0.54–0.79)	<.001	0.61 (0.49–0.76)	<.001	0.58 (0.44–0.77)	<.001	72.0	56.5
Stage IV								
PRRT	Ref.		Ref.		Ref.		21.4	14.3
PORT	1.14 (0.77–1.68)	0.527	1.08 (0.68–1.71)	0.737	0.92 (0.54–1.56)	0.750	37.6	5.8

^*^Cox proportional hazard regression analysis for overall survival and competing risk survival models for breast cancer–specific survival were used (two-sided). Covariates for multivariable/PS matching-adjustment: age, race, Hispanic origin, marital status, place of residence, laterality, tumor grade, stage, LN status, surgery mode, ER status, and PR status. CI = confidence interval; HR = hazard ratio; PS = propensity score; PRRT = preoperative radiotherapy; PORT = postoperative radiotherapy; Ref. = referent.

^†^Multivariate analysis of PS matching-adjusted groups.

The findings concerning DSS were comparable to those from the secondary 1:1 PS matching-adjusted competing risk analyses (Supplementary Table 5).

## DISCUSSION

Our data confirmed the critical role of radiation in the multidisciplinary management of breast cancer [9, 22]. As the use of different sequence patterns for RT and surgery has gained attention in recent clinical trials [23, 24], we analyzed the rate of all the patterns recorded in SEER and found that sequence patterns other than PORT were rarely used, with the exception of in the Utah registry. Treatment trends in Utah shifted abruptly from a median of 98.8% of cases undergoing PRRT before 1988 to a median of 99.0% of cases receiving PORT after 1988. Reasons for this geographical difference as well as the abrupt time variation remain unclear and should be further studied.

Significantly higher risks of both OM and BCM were observed among patients who received PRRT compared to those who underwent PORT. Recent evidence has demonstrated that cancer stem cell (CSC) frequency is correlated not only to outcomes for patients with malignant cancers but also to radiation resistance [25, 26]. After conventional treatment, residual breast tumor cell populations may survive and be enriched with CSCs, resulting in recurrence and reduced long-term survival in breast cancer patients [27–29]. Pathological complete response (pCR) after preoperative therapy leads to visibly reduced tumor size, providing a chance for surgery and improving the rate of complete remission for operable tumors. Nevertheless, the cells that survive RT and are not removed because of the reduced extent of resection are likely to cause the worse outcomes in PRRT than in PORT. Moreover, PRRT may also inhibit the function of hematopoietic system and immune system, which is highly sensitive to radiation (ref), and then may reduce the long-term outcome of patients. In the survival analyses, a strong association was observed between various types of mortality and clinical stage. Significantly worse outcomes were observed for PRRT than PORT in patients diagnosed with stage I, II and III cancer but not stage IV. The small number of patients with stage IV cancer may have led to the non-significant difference between treatments for those patients. These results suggest that further assessment of the long-term treatment efficacy of different modalities of radiation with surgery is warranted and that a less invasive and more restrained and individualized local treatment strategy based on one's probability of local recurrence and mortality risk should be considered.

Moreover, with regard to mortality from causes other than breast cancer, the group undergoing PRRT was more likely to die of cardiovascular disease than the PORT group. Although the increased risks for cardiac mortality of radiotherapy have been well established [30, 31], further investigation into why the PRRT group had higher cardiotoxicity is needed.

One strength of our study is that it is a novelpopulation-based study to investigate the trends in radiation sequence with surgery and to compare the outcomes between PRRT and PORT. Although similar studies [22, 32] have pointed out the benefit of adjuvant radiotherapy after cancer-directed surgery for patients with cancer, such as metastatic gastric cancer and ductal carcinoma *In Situ*. However, the difference in outcomes of sequences of surgery and radiation for treatment of cancer hasn't been systematically addressed. SEER has long employed a consistent algorithm not only to ascertain cause of death but also to ensure that disease-specific survival is determined as accurately as possible [14]. Moreover, advanced statistical methodology to account for the known biases between treatment groups in retrospective data were also applied in our study. Furthermore, our results provide information to guide individual treatment options based on clinical stages and to inform future studies.

There are several limitations to our study. Because unmeasured confounders such as pCR following PRRT and reasons for treatment selection were not available in the SEER database and may have influenced overall results, our results should be interpreted with some caution. To date, whether there is a direct relationship between pCR in neoadjuvant therapy and long-term outcomes is still uncertain [33]. However, if improved pCR is associated with improved long-term survival, differences in outcome between PRRT and PORT may be underestimated for pCR-positive patients in this study. Hence, more prospective studies should be performed to evaluate the survival difference between sequence patterns. Another limitation of our study is the relatively small number of cases included. The geographical characterization and abrupt time variation might have led to bias in treatment outcomes. Finally, although we evaluated the association of treatment with survival endpoints only, it is important to acknowledge that recurrence endpoints and impact on quality of life are also meaningful to patients and thus should be considered in future studies.

## CONCLUSIONS

In conclusion, PORT was still the first option of radiation sequence with surgery between 1991 and 2010 in the United States. Compared with patients who underwent PORT, those who received PRRT had a strikingly worse OS and DSS, indicating the need for a more thoughtful and restrained treatment approach to this disease. Therefore, prospective studies aiming to confirm our findings and tailored sequence modalities for malignant breast cancer are urgently needed.

## SUPPLEMENTARY MATERIALS FIGURES AND TABLES





## Abbreviations

AJCC: American Joint Committee on Cancer
BCM: breast cancer-specific mortality
BCS: breast-conserving surgery
CI: confidence interval
CSC: cancer stem cells
DSS: disease-specific survival
ER: estrogen receptor
HR: hazard ratio
LN: lymph node
OM: overall mortality
OS: overall survival
PRRT: preoperative radiotherapy
PORT: postoperative radiotherapy
pCR: Pathological complete response
SEER: The Surveillance, Epidemiology, and End Results
PS: propensity score
RT: radiotherapy
PR: progesterone receptor
