# Supplementary material for: Trends and outcomes of neoadjuvant radiotherapy compared with postoperative radiotherapy for malignant breast cancer
Source: Oncotarget. 2018 May 11;9(36):24525–36. doi: 10.18632/oncotarget.24313 (PMC5966264; doi:10.18632/oncotarget.24313)
Supplement: Supplementary file 1 [file oncotarget-09-24525-s001.pdf]

Trends and outcomes of neoadjuvant radiotherapy compared with postoperative radiotherapy for malignant breast cancer

SUPPLEMENTARY MATERIALS

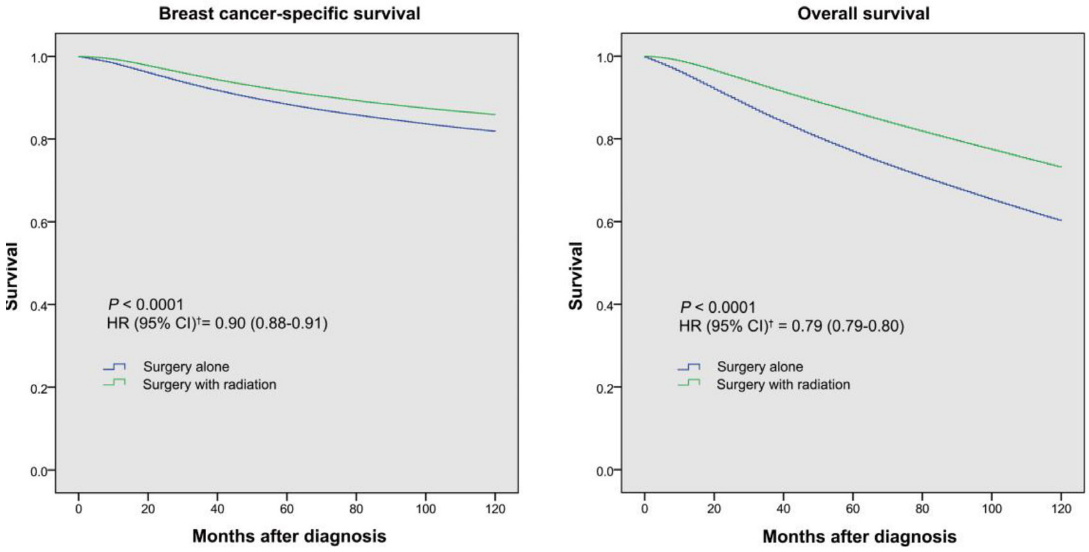

Supplementary Figure 1: OS and BCSS by groups of surgery alone and surgery with radiation in 18 states from 1988 to 2007. †Multivariate analysis adjusted by age, race, Hispanic origin, marital status, place of residence, laterality, tumor grade, stage, LN status, surgery mode, ER status, and PR status. HR = hazard ratio, CI = confidence interval, OS = overall survival, BCSS = breast cancer–specific survival. All P values based on two-sided log-rank test. Left column, breast cancer-specific mortality. Right column, overall survival.

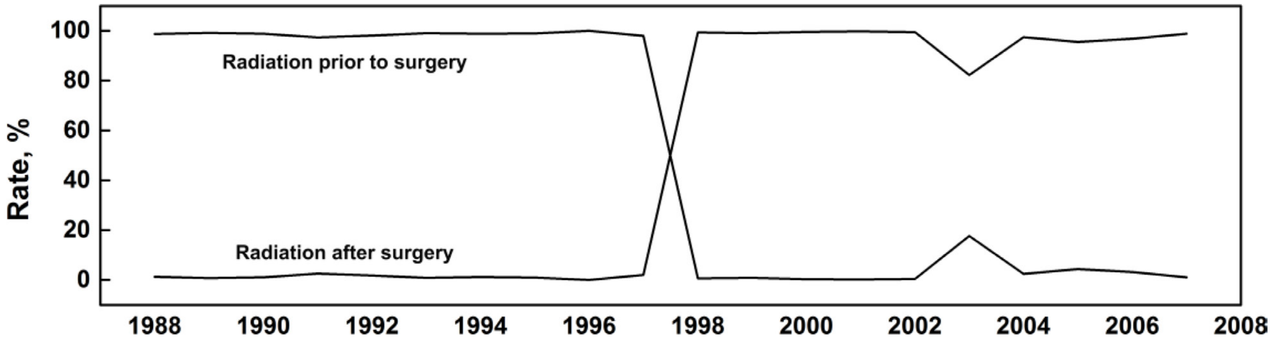

Supplementary Figure 2: Time trends of treatment among breast cancer patients registered in Utah from 1988 to 2007.

Supplementary Table 1: Patient characteristics of SEER cohort diagnosed with breast cancer and underwent surgery in 18 registries from 1988 to 2007 (*n* = 636181 patients)*. See_Supplementary_Table 1

Supplementary Table 2: Original registries data of radiation sequence with surgery in 18 registries from 1973 to 2013 (n = 1111218 patients)*

Registry	No. of Patients (%)								P-value†
	SURG (n = 636807)	PRRT (n = 7644)	PORT (n = 461465)	IntRT (n = 1091)	IntRT+other (n = 490)	RT-SURG-RT (n = 1992)	SURG-RT-SUGR (n = 20)	Unknown (n = 1709)	
Alaska Natives, 1992+	672 (61.6)	3 (0.3)	414 (37.9)	0 (0.0)	0 (0.0)	1 (0.1)	0 (0.0)	1 (0.1)	<.001
Atlanta (metropolitan), 1975+	28442 (57.5)	239 (0.5)	20644 (41.7)	3 (0.0)	0 (0.0)	32 (0.1)	0 (0.0)	90 (0.2)	
California excluding SF/ SJM/LA - 2000+	87088 (53.6)	347 (0.2)	74137 (45.6)	418 (0.3)	138 (0.1)	275 (0.2)	0 (0.0)	187 (0.1)	
Connecticut, 1973+	59395 (63.6)	227 (0.2)	33626 (36.0)	28 (0.0)	5 (0.0)	59 (0.1)	0 (0.0)	59 (0.1)	
Detroit (metropolitan), 1973+	60538 (61.4)	514 (0.5)	37329 (37.9)	54 (0.1)	51 (0.1)	122 (0.1)	2 (0.0)	6 (0.0)	
Greater Georgia - 2000+	25945 (54.8)	191 (0.4)	21007 (44.4)	80 (0.2)	40 (0.1)	29 (0.1)	1 (0.0)	35 (0.1)	
Hawaii, 1973+	13676 (54.2)	90 (0.4)	11413 (45.3)	1 (0.0)	2 (0.0)	18 (0.1)	0 (0.0)	12 (0.0)	
Iowa, 1973+	50376 (66.5)	198 (0.3)	25066 (33.1)	46 (0.1)	26 (0.0)	40 (0.1)	0 (0.0)	30 (0.0)	
Kentucky, 2000+	21587 (55.7)	137 (0.4)	16998 (43.8)	2 (0.0)	0 (0.0)	32 (0.1)	9 (0.0)	9 (0.0)	
Los Angeles, 1992+	68093 (61.2)	379 (0.3)	42419 (38.1)	110 (0.1)	33 (0.0)	174 (0.2)	1 (0.0)	138 (0.1)	
Louisiana, 2000+	19835 (53.4)	209 (0.6)	16207 (43.7)	35 (0.1)	23 (0.1)	694 (1.9)	0 (0.0)	107 (0.3)	
New Jersey, 2000+	44607 (52.8)	293 (0.3)	38876 (46.0)	37 (0.0)	14 (0.0)	42 (0.0)	0 (0.0)	594 (0.7)	
New Mexico, 1973+	19823 (62.1)	146 (0.5)	11666 (36.6)	16 (0.1)	39 (0.1)	175 (0.5)	0 (0.0)	33 (0.1)	
Rural Georgia, 1992+	1057 (63.0)	7 (0.4)	614 (36.6)	0 (0.0)	0 (0.0)	1 (0.1)	0 (0.0)	0 (0.0)	
San Francisco-Oakland SMSA, 1973+	54078 (55.2)	327 (0.3)	42931 (43.8)	139 (0.1)	104 (0.1)	215 (0.2)	0 (0.0)	188 (0.2)	
San Jose-Monterey, 1992+	15080 (49.7)	61 (0.2)	15064 (49.7)	23 (0.1)	11 (0.0)	45 (0.1)	0 (0.0)	55 (0.2)	
Seattle (Puget Sound), 1974+	47601 (51.6)	165 (0.2)	44306 (48.0)	82 (0.1)	4 (0.0)	30 (0.0)	6 (0.0)	84 (0.1)	
Utah, 1973+	18914 (59.3)	4111 (12.9)	8748 (27.4)	17 (0.1)	0 (0.0)	8 (0.0)	1 (0.0)	81 (0.3)	

*LA = Los Angeles; RT = radiotherapy; SF = San Francisco; SJM = San Jose-Monterey; SMSA = standard metropolitan statistical area; SURG = surgery without radiotherapy; PRRT = preoperative radiotherapy; PORT = postoperative radiotherapy; IntRT = intraoperative radiation; IntRT+other = intraoperative radiation with other radiation before/after surgery; RT-SURG-RT = radiation before and after surgery; SURG-RT-SUGR = surgery both before and after radiation; Unknown = sequence unknown, but both were given.
†P values based on Pearson's Chi-square test for categorical (counts, percentage) variables.

Supplementary Table 3: Hazard of OM between groups (unadjusted, multivariable-adjusted, and PS matching-adjusted Cox proportional hazards regression models) in Utah form 1988-2007*

	Unadjusted		Multivariable-adjusted		PS matching-adjusted†		PS matching-adjusted†	
**Characteristic**	**HR (95% CI)**	***P***	**HR (95% CI)**	***P***	**HR (95% CI)**	***P***	**HR (95% CI)**	***P***
Treatment								
PRRT	Ref.		Ref.		Ref.		Ref.	
PORT	0.79 (0.73–0.86)	<.001	0.82 (0.75–0.90)	<.001	0.85 (0.76–0.94)	0.002	0.79 (0.71–0.88)	<.001
Age group, y								
20-39	Ref.		Ref.		Ref.		Ref.	
40-49	0.68 (0.56–0.84)	<.001	0.85 (0.69–1.05)	0.13	0.75 (0.57–0.98)	0.036	0.95 (0.72–1.26)	0.723
50-69	0.97 (0.81–1.16)	0.715	1.26 (1.05–1.52)	0.012	1.09 (0.86–1.39)	0.475	1.42 (1.11–1.80)	0.005
≥70	2.93 (2.45–3.51)	<.001	3.94 (3.27–4.74)	<.001	3.24 (2.56–4.11)	<.001	4.18 (3.27–5.34)	<.001
Married								
Yes	Ref.		Ref.		Ref.		Ref.	
No	1.67 (1.54–1.81)	<.001	1.31 (1.21–1.42)	<.001	1.67 (1.51–1.85)	<.001	1.31 (1.18–1.46)	<.001
Unknown	1.36 (1.00–1.86)	0.050	1.36 (1.00–1.86)	0.052	1.44 (0.90–2.30)	0.126	1.38 (0.87–2.21)	0.174
Grade								
High	Ref.		Ref.		Ref.		Ref.	
Intermediate	1.27 (1.13–1.42)	<.001	1.19 (1.05–1.34)	0.005	1.10 (0.95–1.27)	0.193	1.12 (0.97–1.30)	0.135
Low	1.57 (1.40–1.77)	<.001	1.33 (1.17–1.52)	<.001	1.40 (1.20–1.63)	<.001	1.27 (1.07–1.50)	0.005
Unknown	1.32 (1.14–1.53)	<.001	1.07 (0.92–1.25)	0.358	1.11 (0.92–1.33)	0.275	1.01 (0.83–1.21)	0.949
Stage								
I	Ref.		Ref.		Ref.		Ref.	
II	1.41 (1.28–1.56)	<.001	1.21 (1.06–1.39)	0.005	1.45 (1.27–1.66)	<.001	1.30 (1.09–1.54)	0.003
III	3.17 (2.86–3.51)	<.001	2.26 (1.89–2.71)	<.001	3.46 (3.02–3.96)	<.001	2.63 (2.08–3.31)	<.001
IV	12.34 (10.17–14.98)	<.001	7.89 (6.21–9.96)	<.001	11.11 (8.74–14.11)	<.001	8.22 (6.14–11.01)	<.001
Unknown/other	2.62 (2.30–2.99)	<.001	1.36 (1.11–1.66)	0.003	2.90 (2.47–3.41)	<.001	1.41 (1.10–1.81)	0.007
LN status								
Negative	Ref.		Ref.		Ref.		Ref.	
Positive	2.11 (1.94–2.29)	<.001	1.20 (1.04–1.38)	0.013	2.31 (2.07–2.58)	<.001	1.18 (0.99–1.42)	0.071
Unknown	3.07 (2.72–3.47)	<.001	1.50 (1.24–1.81)	<.001	3.37 (2.91–3.90)	<.001	1.70 (1.34–2.14)	<.001
ER status								
Positive	Ref.		Ref.		Ref.		Ref.	
Negative	1.23 (1.11–1.36)	<.001	1.14 (0.98–1.32)	0.085	1.15 (1.01–1.31)	0.0452	1.09 (0.91–1.31)	0.362
borderline	NA	NA	NA	NA	NA	NA	NA	NA
Unknown/other	1.24 (1.11–1.38)	<.001	1.47 (0.81–2.67)	0.200	1.30 (1.11–1.52)	0.001	1.07 (0.52–2.21)	0.854
PR status								
Positive	Ref.		Ref.		Ref.		Ref.	
Negative	1.34 (1.22–1.47)	<.001	1.26 (1.11–1.43)	<.001	1.33 (1.19–1.50)	<.001	1.36 (1.16–1.58)	<.001
borderline	1.36 (1.02–1.83)	0.038	1.20 (0.89–1.61)	0.238	1.11 (0.76–1.62)	0.586	1.07 (0.73–1.57)	0.728
Unknown/other	1.30 (1.17–1.45)	<.001	0.75 (0.42–1.35)	0.343	1.38 (1.18–1.62)	<.001	1.20 (0.59–2.45)	0.610
Surgery, primary site								
BCS	Ref.		Ref.		Ref.		Ref.	
Mastectomy	2.24 (2.06–2.43)	<.001	1.49 (1.34–1.66)	<.001	2.23 (2.01–2.49)	<.001	1.37 (1.20–1.57)	<.001
Unknown/other	NA	NA	NA	NA	NA	NA	NA	

*PS: propensity score; Ref.: reference group; LN = Lymph node; ER = estrogen receptor; PR = progesterone receptor; PRRT = preoperative radiotherapy; PORT = postoperative radiotherapy; CI = confidence interval; HR = hazard ratio; OM = overall mortality. All tests are two-sided. Covariates used for multivariable-adjusted analysis: age, marital status, tumor grade, stage, LN status, surgery mode, ER status, and PR status. NA: not applicable.

†univariable-adjusted Cox proportional hazard modelling evaluated the HRs of 1:1 matched groups.

‡Multivariable-adjusted Cox proportional hazard modelling evaluated the HRs of 1:1 matched groups.

Supplementary Table 4: Hazard of BCM between groups (unadjusted, multivariable-adjusted, and PS matching-adjusted Cox proportional hazards regression models) in Utah form 1988-2007*

	Unadjusted		Multivariable-adjusted		PS matching-adjusted†		PS matching-adjusted‡	
**Characteristic**	**HR (95% CI)**	***P***	**HR (95% CI)**	***P***	**HR (95% CI)**	***P***	**HR (95% CI)**	***P***
Treatment								
PRRT	Ref.		Ref.		Ref.		Ref.	
PORT	0.80 (0.71–0.91)	<.001	0.71 (0.62–0.81)	<.001	0.80 (0.68–0.94)	0.007	0.69 (0.59–0.82)	<.001
Age group, y								
20-39	Ref.		Ref.		Ref.		Ref.	
40-49	0.63 (0.51–0.79)	<.001	0.94 (0.75–1.18)	0.599	0.65 (0.48–0.88)	0.005	1.05 (0.78–1.42)	0.743
50-69	0.51 (0.42–0.63)	<.001	0.84 (0.68–1.03)	0.090	0.54 (0.42–0.71)	<.001	0.89 (0.68–1.17)	0.411
≥70	0.68 (0.55–0.85)	0.001	1.34 (1.07–1.69)	0.011	0.66 (0.50–0.88)	0.004	1.35 (1.00–1.82)	0.047
Married								
Yes	Ref.		Ref.		Ref.		Ref.	
No	1.23 (1.08–1.39)	0.001	1.13 (0.99–1.29)	0.063	1.16 (0.98–1.36)	0.087	NA	NA
Unknown	1.59 (1.05–2.41)	0.028	1.75 (1.15–2.66)	0.008	1.65 (0.88–3.08)	0.120	NA	NA
Grade								
High	Ref.		Ref.		Ref.		Ref.	
Intermediate	3.12 (2.40–4.05)	<.001	2.06 (1.58–2.69)	<.001	2.52 (1.82–3.50)	<.001	1.85 (1.32–2.58)	<.001
Low	6.01 (4.64–7.80)	<.001	2.75 (2.09–3.62)	<.001	5.13 (3.71–7.11)	<.001	2.52 (1.78–3.56)	<.001
Unknown	3.10 (2.27–4.22)	<.001	1.79 (1.30–2.46)	<.001	2.42 (1.65–3.54)	<.001	1.65 (1.12–43)	0.012
Stage								
I	Ref.		Ref.		Ref.		Ref.	
II	2.86 (2.40–3.42)	<.001	1.78 (1.42–2.24)	<.001	2.95 (2.33–3.73)	<.001	1.92 (1.43–2.57)	<.001
III	9.29 (7.88–10.95)	<.001	4.16 (3.15–5.49)	<.001	9.73 (7.82–12.10)	<.001	4.91 (3.42–7.04)	<.001
IV	38.06 (29.95–48.37)	<.001	17.82 (12.94–24.56)	<.001	34.15 (25.24–46.20)	<.001	18.24 (12.15–27.37)	<.001
Unknown/other	2.91 (2.21–3.83)	<.001	1.79 (1.25–2.56)	0.001	3.01 (2.15–4.22)	<.001	1.91 (1.22–2.98)	0.005
LN status								
Negative	Ref.		Ref.		Ref.		Ref.	
Positive	4.64 (4.07–5.30)	<.001	1.34 (1.09–1.66)	0.006	5.02 (4.22–5.97)	<.001	1.31 (1.00–1.72)	0.052
Unknown	3.41 (2.73–4.26)	<.001	1.46 (1.08–1.98)	0.015	3.32 (2.52–4.38)	<.001	1.68 (1.14–2.47)	0.008
ER status								
Positive	Ref.		Ref.		Ref.		Ref.	
Negative	1.97 (1.72–2.26)	<.001	1.25 (1.02–1.54)	0.032	1.91 (1.59–2.30)	<.001	1.27 (0.97–1.67)	0.078
borderline	NA	NA	NA	NA	NA	NA	NA	NA
Unknown/other	1.46 (1.22–1.74)	<.001	1.12 (0.50–2.54)	0.778	1.53 (1.18–1.97)	0.001	0.85 (0.34–2.11)	0.721
PR status								
Positive	Ref.		Ref.		Ref.		Ref.	
Negative	2.02 (1.77–2.30)	<.001	1.42 (1.17–1.72)	<.001	2.00 (1.68–2.37)	<.001	1.35 (1.06–1.71)	0.002
borderline	1.88 (1.24–2.86)	0.003	1.37 (0.89–2.09)	0.150	1.20 (0.64–2.25)	0.572	0.86 (0.44–1.69)	0.934
Unknown/other	1.60 (1.34–1.91)	<.001	1.04 (0.47–2.30)	0.929	1.71 (1.33–2.20)	<.001	1.50 (0.62–3.65)	0266
Surgery, primary site								
BCS	Ref.		Ref.		Ref.		Ref.	
Mastectomy	4.64 (4.13–5.23)	<.001	1.70 (1.46–1.97)	<.001	4.70 (4.014–5.50)	<.001	1.62 (1.33–1.98)	<.001
Unknown/other	NA	NA	NA	NA	NA	NA	NA	NA
Living								
Urban large	Ref.		Ref.		Ref.		NA	
Urban small	0.89 (0.75–1.05)	0.160	0.93 (0.79–1.10)	0.419	0.78 (0.61–1.00)	0.051	0.80 (0.63–1.02)	0.074
Rural	1.31 (1.03–1.68)	0.030	1.10 (0.86–1.41)	0.456	1.41 (1.03–1.93)	0.030	0.97 (0.71–1.34)	0.864

*PS: propensity score; Ref.: reference group; LN = Lymph node; ER = estrogen receptor; PR = progesterone receptor; PRRT = preoperative radiotherapy; PORT = postoperative radiotherapy; CI = confidence interval; HR = hazard ratio; BCM = breast cancer–specific mortality. All tests are two-sided. Covariates used for multivariable-adjusted analysis: age, marital status, place of residence, tumor grade, stage, LN status, surgery mode, ER status, and PR status. NA: not applicable.

†univariable-adjusted Cox proportional hazard modelling evaluated the HRs of 1:1 matched groups.

‡Multivariable-adjusted Cox proportional hazard modelling evaluated the HRs of 1:1 matched groups.

Supplementary Table 5: HRs comparing DSS between PORT group and PRRT group with 1:1 PS matching–adjusted competing risk analyses*

Stage	Breast cancer–specific survival		Other malignant disease–specific mortality		Cardiovascular disease–specific mortality	
	**HR (95% CI)**	***P***	**HR (95% CI)**	***P***	**HR (95% CI)**	***P***
Entire cohort	0.78 (0.67–0.92)	0.003	0.89 (0.65–1.21)	0.460	0.64 (0.50–0.81)	<.001
Stage I	0.69 (0.48–1.01)	0.054				
Stage II	0.77 (0.54–1.09)	0.140				
Stage III	0.62 (0.47–0.81)	<.001				
Stage IV	0.94 (0.57–1.56)	0.820				

*CI = confidence interval; HR = hazard ratio; PS = propensity score; PRRT = preoperative radiotherapy; PORT = postoperative radiotherapy; DSS = disease-specific survival.
