# Supplementary material for: Trends and outcomes of neoadjuvant radiotherapy compared with postoperative radiotherapy for malignant breast cancer
Source: Oncotarget. 2018 May 11;9(36):24525–36. doi: 10.18632/oncotarget.24313 (PMC5966264; doi:10.18632/oncotarget.24313)
Supplement: Supplementary file 2 [file oncotarget-09-24525-s002.docx]

## Supplementary Table 1: Patient characteristics of SEER cohort diagnosed with breast cancer and underwent surgery in 18 registries from 1988 to 2007 (n = 636181 patients)*

| Characteristic | No. of Patients (%) | | | *P*-value† |
| --- | --- | --- | --- | --- |
|  | Non-RT Group | RT Group | Total |  |
| Number of patients | 351768 | 284413 | 636181 |  |
| Race | | | | |
| White | 293950 (83.6) | 240173 (84.4) | 534123 (84.0) | <.001 |
| Black | 32502 (9.2) | 23876 (8.4) | 56378 (8.9) |  |
| Other/unknown | 25316 (7.2) | 20364 (7.2) | 45680 (7.2) |  |
| Hispanic | | | | |
| Yes | 26656 (7.6) | 21597 (7.6) | 48253 (7.6) | 0.813 |
| No | 325112 (92.4) | 262816 (92.4) | 587928 (92.4) |  |
| Age group, y | | | | |
| 20-39 | 19307 (5.5) | 17339 (6.1) | 36646 (5.8) | <.001 |
| 40-49 | 58133 (16.5) | 56141 (19.7) | 114274 (18.0) |  |
| 50-69 | 145542 (41.4) | 141641 (49.8) | 287183 (45.1) |  |
| ≥70 | 128786 (36.6) | 69292 (24.4) | 198078 (31.1) |  |
| Married | | | | |
| Yes | 183147 (52.1) | 170467 (59.9) | 353614 (55.6) | <.001 |
| No | 155165 (44.1) | 106807 (37.6) | 261972 (41.2) |  |
| Unknown | 13456 (3.8) | 7139 (2.5) | 20595 (3.2) |  |
| Laterality | | | | |
| Unilateral | 351274 (99.9) | 284261 (99.9) | 635535 (99.9) | <.001 |
| Bilateral | 494 (0.1) | 152 (0.1) | 646 (0.1) |  |
| Grade | | | | |
| High | 52691 (15.0) | 54790 (19.3) | 107481 (16.9) | <.001 |
| Intermediate | 120952 (34.4) | 106119 (37.3) | 227071 (35.7) |  |
| Low | 115348 (32.8) | 92792 (32.6) | 208140 (32.7) |  |
| Unknown | 62777 (17.8) | 30712 (10.8) | 93489 (14.7) |  |
| Stage | | | | |
| I | 148920 (42.3) | 144688 (50.9) | 293608 (46.2) | <.001 |
| II | 121523 (34.5) | 79778 (28.1) | 201301 (31.6) |  |
| III | 39239 (11.2) | 41358 (14.5) | 80597 (12.7) |  |
| IV | 8116 (2.3) | 4957 (1.7) | 13073 (2.1) |  |
| Unknown/other | 33970 (9.7) | 13632 (4.8) | 47602 (7.5) |  |
| Tumor size, mm | | | | |
| ≤20 | 58760 (16.7) | 63661 (22.4) | 122421 (19.2) | <.001 |
| 21-50 | 32290 (9.2) | 25764 (9.1) | 58054 (9.1) |  |
| ＞50 | 5860 (1.7) | 6305 (2.2) | 12165 (1.9) |  |
| Unknown | 254858 (72.5) | 188683 (66.3) | 443541 (69.7) |  |
| LN status | | | | |
| Negative | 192316 (54.7) | 170699 (60.0) | 363015 (57.1) | <.001 |
| Positive | 104441 (29.7) | 88386 (31.1) | 192827 (30.3) |  |
| Unknown | 55011 (15.6) | 25328 (8.9) | 80339 (12.6) |  |
| ER status | | | | |
| Positive | 205590 (58.4) | 196526 (69.1) | 402116 (63.2) | <.001 |
| Negative | 62368 (17.7) | 52689 (18.5) | 115057 (18.1) |  |
| borderline | 1499 (0.4) | 886 (0.3) | 2385 (0.4) |  |
| Unknown/other | 82311 (23.4) | 34312 (12.1) | 116623 (18.3) |  |
| PR status | | | | |
| Positive | 169941 (48.3) | 165920 (58.3) | 335861 (52.8) | <.001 |
| Negative | 90706 (25.8) | 77969 (27.4) | 168675 (26.5) |  |
| borderline | 2686 (0.8) | 1851 (0.7) | 4537 (0.7) |  |
| Unknown/other | 88435 (25.1) | 38673 (13.6) | 127108 (20.0) |  |
| Surgery, primary site | | | | |
| BCS | 96214 (27.4) | 228019 (80.2) | 324233 (51.0) | <.001 |
| Mastectomy | 254311 (72.3) | 55899 (19.7) | 310210 (48.8) |  |
| Unknown/other | 1243 (0.4) | 495 (0.2) | 1738 (0.3) |  |
| Living | | | | |
| Urban large | 279051 (79.3) | 234095 (82.3) | 513146 (80.7) | <.001 |
| Urban small | 60089 (17.1) | 42323 (14.9) | 102412 (16.1) |  |
| Rural | 7510 (2.1) | 4355 (1.5) | 11865 (1.9) |  |
| Unknown | 5118 (1.5) | 3640 (1.3) | 8758 (1.4) |  |
| Year‡ | | | | |
| 1973-1983 | 55638 (15.8) | 22697 (8.0) | 78335 (12.3) | <.001 |
| 1984-1993 | 64517 (18.3) | 45164 (15.9) | 109681 (17.2) |  |
| 1994-2003 | 106126 (30.2) | 94504 (33.2) | 200630 (31.5) |  |
| 2004-2013 | 125487 (35.7) | 122048 (42.9) | 247535 (38.9) |  |
| Status | | | | |
| Dead breast | 61820 (17.6) | 41174 (14.5) | 102994 (16.2) | <.001 |
| Dead other | 107398 (30.5) | 52063 (18.3) | 159461 (25.1) |  |
| Alive | 182550 (51.9) | 191176 (67.2) | 373726 (58.7) |  |

* BCS = breast-conserving surgery; LN = Lymph node; ER = estrogen receptor; PR = progesterone receptor; RT = .radiotherapy.

† *P* values based on Pearson’s Chi-square test for categorical (counts, percentage) variables.

‡ Numbers and row percentages.
